# Understanding the interface between European wild boar (*Sus scrofa*) and domestic pigs (*Sus scrofa domesticus*) in Sweden through a questionnaire study

**DOI:** 10.1186/s13028-023-00705-x

**Published:** 2023-09-22

**Authors:** Linda Ernholm, Karl Ståhl, Aleksija Neimanis, Stefan Widgren, Susanna Sternberg-Lewerin

**Affiliations:** 1 Department of Biomedical Sciences and Veterinary Public Health, Swedish University of Agricultural Sciences (SLU), 750 07 Uppsala, Sweden; 2 Department of Disease Control and Epidemiology, National Veterinary Institute (SVA), 751 89 Uppsala, Sweden; 3 Department of Pathology and Wildlife Diseases, National Veterinary Institute (SVA), 751 89 Uppsala, Sweden

**Keywords:** African swine fever, Disease transmission, Wildlife-livestock interface

## Abstract

**Background:**

In recent years, the wildlife/livestock interface has attracted increased attention due to disease transmission between wild and domestic animal populations. The ongoing spread of African swine fever (ASF) in European wild boar (*Sus scrofa*) emphasize the need for further understanding of the wildlife/livestock interface to prevent disease spill-over between the wild and domestic populations. Although wild boar may also act as a potential source for other infectious disease agents, ASF is currently the most severe threat from wild boar to domestic pigs. To gather information on the wild boar situation at commercial pig producing farms in Sweden, a digital questionnaire survey was distributed through the animal health services.

**Results:**

Most pigs produced for commercial purposes in Sweden are raised without outdoor access. Of the 211 responding pig producers, 80% saw wild boar or signs of wild boar activity in the vicinity of their farm at least once during the year. Observations were significantly correlated with geographical region, but there was no correlation between farm characteristics (farm size, main type of production, outdoor access) and observed wild boar presence or proximity. However, a reported higher frequency of wild boar observations was positively correlated with the observations being made in closer proximity to the farm.

Hunting and strategic baiting were the most common mitigation strategies used to keep wild boar at bay. Of the 14 farms raising pigs with outdoor access, 12 responded that these pigs could be raised solely indoors if needed.

Pigs with outdoor access are required to be fenced in, but double fencing in these outdoor pig enclosures was not practiced by all. A perimeter fence surrounding any type of pig farm was very rare. More than half of the producers that grew crops with intended use for pigs reported crop damage by wild boar.

**Conclusion:**

This study shows that although pigs raised for commercial purposes in Sweden are, to a large extent, kept indoors the potential for indirect contact with wild boar exists and must be considered. Variable local situations regarding wild boar abundance may require an adaptive approach regarding biosecurity efforts.

**Supplementary Information:**

The online version contains supplementary material available at 10.1186/s13028-023-00705-x.

## Background

In recent years, the wildlife/livestock interface has attracted increased attention due to disease transmission between wild and domestic animal populations [1, 2]. Recurrent seasonal epidemics of highly pathogenic avian influenza in wild birds, and the ongoing spread of African swine fever (ASF) in European wild boar (*Sus scrofa*) are two examples of disease events that demonstrate the need for further understanding of the wildlife/livestock interface to prevent disease spill-over between the wild and domestic populations. Wild boar may also act as a potential source for other infectious disease agents such as *Salmonella*, *Mycoplasma hyopneumoniae* and *Toxoplasma gondii* [3]. However, ASF is currently the most severe threat from wild boar to domestic pig populations.

In domestic pigs (*Sus scrofa domesticus*) and European wild boar (*Sus scrofa),* infection with ASF virus (ASFV) typically causes a contagious haemorrhagic fever with high case fatality rate [4]. The virus can be spread through direct contact with infected animals or carcasses or indirectly through contaminated fomites, transports, and materials such as feed or bedding [5–7]. ASFV of genotype II was introduced from the African continent to Georgia in 2007, causing the current epidemic in Europe, Asia, and parts of Oceania. North America was added to the list of affected continents in 2021 following incursion into the Dominican Republic and Haiti [8].

ASFV is not zoonotic. Nevertheless, the disease has devastating effects on animal health and welfare, and far-reaching consequences for farmers, stakeholders, and trade in affected countries. In Europe and most other countries in the global north, ASF outbreaks in domestic pigs will result in whole-herd slaughter and application of movement restrictions for pigs and pig products with potential trade consequences for the whole country [9].

Long distance translocations of ASFV, attributed to human activities, have led to unpredictable introductions of the virus to wild boar populations far from known infected areas [10]. In a globalized world, with ASFV present in more countries and on more continents than ever before, the risk of human activities moving infected meat or contaminated products increases [11]. Sweden is currently free from ASF and does not share a land border with any presently affected country. Therefore, the most plausible scenario for a virus introduction to Sweden is through human activities exposing wild boar to ASFV through contaminated objects or infected pork products. This, and subsequent spread to domestic pigs, is feared by Swedish pig producers.

Similar to many other European countries, Sweden has experienced a substantial increase in wild boar abundance during the last two decades. This is reflected in hunting statistics with reports of less than 400 wild boar shot during hunting in 1990, close to 5000 in the year 2000 and just above 160,000 in 2020 [12]. Likewise, the amount of crops damaged by wildlife doubled from 2014 to 2020, with wild boar causing more than 50% of the reported damage done to cereals and forage [13]. Wild boars are present in the southern half of Sweden and the geographical distribution of the species overlaps with the major pig production areas. A study from 2013 describes presence of wild boar within 10 km in 65% of 60 Swedish farrow-to-finish farms [14]. Even though direct contact between wild boars and domestic pigs can be avoided through use of fencing and housing, routes for indirect pathways and consequences of biosecurity breaches may be associated with having wild boars close to pig farms. Therefore, the presence of infected wild boars around farms is a risk factor for infection of domestic pigs with ASFV [7].

Pigs raised for commercial purposes are generally kept indoors. Even though there is an increasing interest and demand for pork from organic production where pigs have outdoor access, less than 3% of the produced pigs in Sweden are raised under these conditions [15]. Swedish animal welfare legislation requires that all pigs have access to materials to manipulate for enrichment purposes and straw is often the material of choice. Sows are kept in groups during their dry period, often on deep litter straw bedding. Sometimes these groups are housed in a well-ventilated barn with large doors or sliding wall sections that can be opened during suitable weather conditions while the animals remain inside. Even though these pigs are still considered to be kept indoors, these more open barns present an opportunity for direct contact with wild boar, should they approach the building.

No detailed study on the wildlife/livestock interface focused on wild boar and commercial pig production has previously been done in Sweden. Understanding of this interface is needed for informed and relevant policy making, creation of biosecurity strategies for contact mitigation and for effective disease prevention and control. The aim of this study was to investigate the possible direct and indirect contact routes between domestic pigs and wild boars in Sweden.

## Methods

A cross-sectional study design was employed. An electronic questionnaire was developed in the tool Netigate (Netigate AB, Stockholm, Sweden). The questionnaire was distributed by email to all pig producers in Sweden affiliated to one of the following pig health organisations: Farm and animal health (FAH), Lundens animal healthcare (LAH), and the district veterinary organisation’s pig animal health service (DV). Together these organisations cover 90–95% of the commercial pig producers in Sweden. Pig farms of all common production types and levels of outdoor access are affiliated to the services: farrow-to-finish, specialized fattening, and specialized piglet producers as well as breeding and gilt-producing herds. The invitation to participate and the link to the online questionnaire was sent to 1003 recipients. The link was sent together with information about the study including that participation was voluntary, all answers were anonymous, and that data would only be presented in an aggregated form to avoid identification of individual respondents. The questionnaire was set so that each respondent could only reply once.

To encourage participation, the survey was introduced at a conference for commercial pig producers before distribution. Three weeks after the link to the online questionnaire was made available to the producers, the study was mentioned on two websites, one targeting pig producers and one general agricultural media site, which acted as a reminder to participate. The questionnaire was available from November 15, 2019, to January 31, 2020. Two weeks prior to closing the survey, a reminder was sent out by email to all who had received the original link.

### Data collection

The questionnaire had 19 closed questions regarding husbandry, mitigation strategies and wild boar observations. In addition, there were five free text fields for comments. A translated version of the closed questions in the questionnaire is included in Additional file 1.

The questions were related to four areas:

- Farm characteristics (geographical region, farm size, main type of production and housing, including outdoor access)
- Mitigation strategies in use to prevent contacts with wild boar (hunting activities, fences, use of strategic bait feeding, as well as the possibility for closed housing of pigs with outdoor access)
- Risk factors for indirect contact (water source usage, crop damage, and hunting practises)
- Observations of wild boar or their activities (seasonality, distance in relation to pig housings, observations in relation to buildings not housing pigs, and occurrence of hybrid litters.)

The questions on wild boar activities combined direct observations of wild boar and observations of signs of their activity, as direct observations are rare and the focus was on how close the animals came, regardless of how they were observed.

No question or commentary field required an answer for progression through the survey.

The questionnaire was developed in collaboration with pig health veterinarians from the three pig health organisations previously mentioned (FAH, LAH, and DV), a pig health expert at the National Veterinary Institute (SVA) and a representative from the Federation of Swedish Farmers (LRF).

### Data management

Data was exported from the survey tool in excel format. Further data handling including cleaning, analysis, and statistical calculations was done in the statistical program R, R Core Team, 2019 [16].

Control of duplicate answers was done by comparing the answers to a select set of questions (postal area, number of pigs, pig housing and mitigation strategies).

When the response option ‘other, please specify’ was used for clarification purposes of a given option only, and not to provide a different alternative, the answers were recoded into the relevant response options.

### Statistical analysis

The geographical representativity of the respondents compared to the target population was assessed by proportional testing. Respondents were asked to provide the first two digits in their postal code which was further aggregated on the European regional level, according to Nomenclature of Territorial Units for Statistics (NUTS 2), of which there are 8 in Sweden. The aggregated responses were then compared to the number of pig enterprises registered in the respective region [15].

To evaluate farm characteristics that may affect the level of wild boar observations done, the variables geographical region, main type of production, farm size, and degree of outdoor contact were selected. Further analysis included whether farm characteristics were associated with the frequency of wild boar observations, or the distance at which these observations were done. Farm location was assessed on NUTS 2 level. Each farm was categorized by size following the size categories used by Pettersson et al., regarding Swedish pig production. For sows, the size categories correspond to the following numbers: ‘small’ (< 100), ‘medium’ (100–400) and ‘large’ (> 400) by number of sows per year. For fattening pigs size categories correspond to the following numbers: ‘small’ (< 5000), ‘medium’ (5000–10000) and ‘large’ (> 10000) fattening pigs produced per year [17]. Integrated farms keeping both categories of animals (sows and fattening pigs) were classified based on whichever category was the largest. Farms were assessed for the level of outdoor access present and classified as ‘outdoor access’ if pigs were allowed to leave the building to go outside (inside a fenced area) or as ‘conventional’ if pigs were held inside in closed buildings. ‘Open wall sections’ stipulates a third category of outdoor contact where the pig housing is very well ventilated through slightly permeable walls or by the use of gates in opened wall segments, keeping the pigs inside the designated building.

Categorical variables were assessed for independence using chi square test or, when there were less than five observations in any group, Fisher’s exact test. To compare medians of a numerical variable by levels of a categorical variable, Kruskal–Wallis rank sum test was used.

### Graphics

Maps were produced in the software R, R Core Team, 2019 [16], using data of registered pig enterprises per region in 2020, obtained from the Swedish Board of Agriculture’s official statistics database [15]. Wild boar abundance was illustrated by the number of wild boars shot per 1000 hectares (10 km^2^) for the hunting year 2019/2020 [12].

## Results

**Fig. 1:**
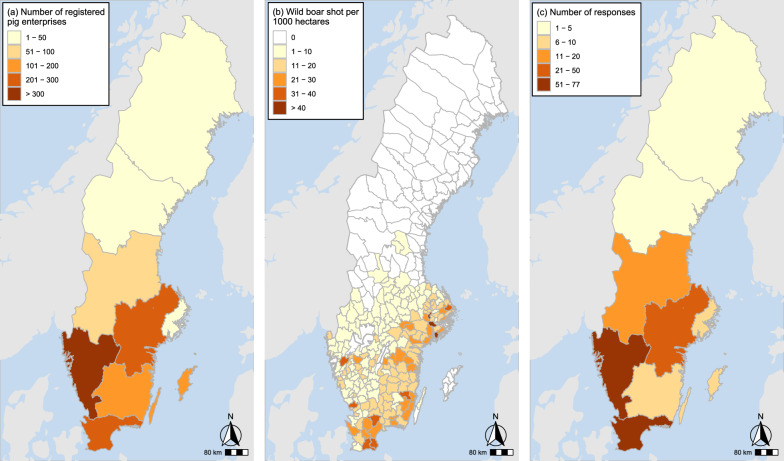
Location of questionnaire respondents in relation to the population of domestic pigs and wild boars. **a** Geographical distribution of pig enterprises on European regional level, NUTS2. **b** Wild boars shot per 1000 hectares (10 km^2^) on the level of regional hunting divisions. **c** The number of questionnaire respondents on European regional level, NUTS2

Of the 1003 invitations sent out to pig producers, 211 (21.0%) submitted a response to the questionnaire. Most of the responses, 83.9% (177/211) were received within the first 10 days. The geographical assessment of response coverage showed that the pig producers in the two most northern regions, as well as the region ‘Småland and the islands’ in the south-east, were slightly less represented in comparison with other regions (Fig. 1). However, with regards to wild boar abundance, all regions were deemed to be sufficiently represented for the purpose of the study (Fig. 1).

The main types of production and farm size among respondents are summarized in Table 1. Ten respondents used the ‘other, please specify’ option for main production type. Based on their specified comment, two respondents belonged to one of the available options and were recoded accordingly. Of the eight remaining in the ‘other’ category, five specified being a sow pool central unit, and the other three were small producers (less than five sows) with outdoor access.

**Table 1:** Main type of production of 206 Swedish pig producers responding to a questionnaire on wild boar presence

Main type of production	Number of respondents	Category of pigs	Number of animals		
			Min	Max	Median (IQR^a^)
Farrow-to-finish	76	year sows^b^	10	950	250 (120–330)
		finishers per year	200	25000	5600 (2800–8000)
Specialized piglet	54	year sows^b^	9	3000	300 (129–500)
		finishers per year^c^	10	6500	135 (80, 425)
Specialized fattening	64	finishers per year	600	47500	3500 (2500–6000)
Breeding/gilt	4	year sows^b^	110	400	–^d^
		finishers per year^c^	2500	6500	–^d^
‘Other’	8	year sows^b^	2	3160	–^d^
		finishers per year	2	300	–^d^

^a^Inter-quartile range (25–75% percentiles)

^b^Sows in production, per year

^c^20 of the 54 specialized piglet producers, and 3 of the 4 breeding/gilt producing herds also produced finishers

^d^Includes diverse categories or few responses making it unsuitable for a median value

### Housing and outdoor access

Of the 211 respondents, 201 provided information about the type of housing. Of these 201 respondents, 194 (96.5%) chose ‘conventional pens or group pens indoors’, 33 (16.4%) ‘pens or group pens in well-ventilated barn with open doors or sliding wall sections’, and 16 (8.0%) ‘outdoor access behind fence/electrical fence’. Four respondents chose ‘other, please specify’, but their comments allowed them to be placed in one or a combination of the given options. As the question allowed for more than one alternative, the percentages add up to more than 100.

### Water source

The two questions regarding source of drinking water for the pigs or for cleaning purposes in pig houses were answered by 197 and 192 respondents, respectively. Water from a well was by far the most common with 158 (80.2%) respondents using this source for drinking water and 156 (81.3%) for cleaning purposes. A single respondent replied using a naturally occurring open water source (such as stream or lake) for drinking water and three respondents indicated the use of such open water source for cleaning purposes. The remaining respondents used municipal water, 38 (19.3%) for drinking and 33 (17.2%) for cleaning.

### Wild boar observations

**Fig. 2:**
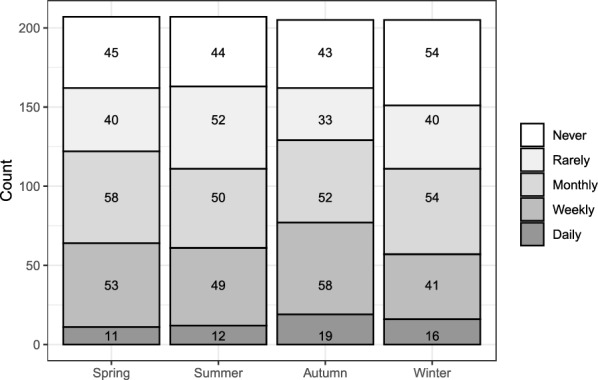
Seasonal wild boar activity in the vicinity of Swedish pig holdings. Frequency of seasonal observations of wild boars or wild boar activity in the vicinity of pig holdings as stated by Swedish pig producers (n = 207)

The question asked for observations of wild boar or their activities during each of the four seasons. Of the 211 respondents, 207 replied to this question and of these 204 answered for all four seasons while three answered for two or three seasons (Fig. 2). Of the 207 responses, 167 (80%) answered that they had seen wild boars or signs of wild boar activity in the vicinity of their pig holding at least once during the year.

As the frequency of wild boar observations did not differ significantly between seasons (P = 0.26), an average observation level per farm was calculated and further classified into three categories, ‘daily to weekly’ (n = 79), ‘monthly to rarely’ (n = 85), or ‘never’ (n = 40), which were used for subsequent analyses of association with farm characteristics.

### Wild boar observations, distance

Respondents who reported wild boar observations were asked to provide information on proximity of observations to their premises. Of the respondents, 114 answered to the question on the shortest distance from different pig holding buildings where they had observed wild boar or wild boar activity. One respondent had contradictory responses regarding distance and observations and was therefore excluded from these results. The responses are summarized in Table 2.

**Table 2:** Distance of wild boar observations to types of pig housing, reported by Swedish pig producers (n = 113)

Type of building	Number of respondents:	Distance of wild boar observations, in meters		
		Min	Max	Median (IQR), meters
Outdoor climate barn/sliding wall sections	29	1	1000	50 (20, 150)
Conventional pig houses	102	1	1000	55 (20, 200)
Pigs with outdoor access, fenced	10	0	200	100 (20, 100)

As more than one type of pig housing may be present on a pig farm, the total number of responses exceed the number of respondents replying to this question

There was no significant difference in the median observed distance between the types of pig housings (P = 0.84), hence only the closest distance reported by each respondent was used in subsequent analyses.

Of the 211 respondents, 192 replied to the question regarding wild boar in the vicinity of other buildings than pig housings on their premises. All but one respondent chose a single answer only, even if the question allowed for multiple answers. Twenty-five of the 192 respondents reported no wild boar presence in the area, 140 stated that wild boars were present in the surroundings, but did not get close to any buildings not housing pigs. Ten respondents indicated that wild boars got close to feed storages and 18 selected ‘other’. One respondent chose both ’other ‘ and ’feed storage’.

### Crop damage

All 211 respondents replied to the question of crop damage by wild boar, 20 stated that they did not grow crops for pigs. Of the 191 farmers that were growing crops, 69 (36.1%) answered that they had not experienced wild boar damage during the growth season of 2019. The remaining 122 (63.9%) reported wild boar damage in grain crops and 19 of them had experienced wild boar damage in other crops as well, mainly protein crops such as peas or field beans, and/or grass and forage crops.

### Hybrid litters

None of the 208 respondents to the question about hybrid litters indicated that there had been a suspected hybrid litter between wild boar and domestic pig in the last 12 months.

### Mitigation strategies and protective measures used to prevent wild boar contact

The responses to the question on mitigation measures are shown in Table 3. Five respondents did not answer this question.

**Table 3:** Mitigation strategies used by responding pig producers to avoid contact with wild boar

Measure	N
Hunting of wild boar in the area	126 (61.2%)
‘Strategic feeding’, baiting off-site	31 (15.0%)
‘Other’	11 (5.3%)
Double fence around pigs with outdoor access^a, b^	4 (1.9%)
Perimeter fence around production site^a^	4 (1.9%)
Nothing	69 (33.5%)

Measures used to avoid wild boar presence at pig production holdings in Sweden as stated by the producers (n = 206)

^a)^ One respondent had both types of fences, the remaining three in each fence category had either perimeter fence or double fence around the outdoor pig enclosure

^b)^ Sixteen producers of pigs with outdoor access replied to the question, hence 25% of relevant producers replied having a double fence

Multiple choices were allowed, hence the total numbers in Table 3 add up to more than 206 or 100%. The option of ‘other’ regarding mitigation strategies was, when specified, either an explanation of why there were no mitigation strategies in place, including being located in a northern region where wild boars are not present, pigs kept in an indoor setting only or the producer did not possess the hunting rights for the land in question. The alternative ‘other’ was also used to make clarifying comments regarding already selected options.

A question about whether all pigs on the farm could be raised indoors only, in case of restrictions imposed during a disease outbreak, was answered by 14 of the 16 respondents who had pigs with outdoor access. Twelve responded that they could raise the pigs exclusively indoors, and two answered that they could not, due to limited space or lack of suitable housing.

Regarding the respondent’s own hunting activities, 176 replied to this question of which 116 (65.9%) said they did not hunt wild boar. The remaining 60 (34.1%) did hunt wild boar in Sweden, and six replied also travelling abroad for wild boar hunting. Regarding the hunting activities of any employees in contact with the pigs there were 175 responses of which 133 (76.0%), replied they had no employee in contact with the pigs who was engaged in hunting of wild boar, 38 (21.7%) replied that employees did hunt in Sweden of which one respondent indicated that employee(s) were also engaged in hunting activities abroad. Four respondents (2.3%) stated that they did not know their employees’ hunting habits.

In the univariable analysis of the farm characteristics geographical region (P < 0.01), main type of production (P = 0.96), farm size (P = 0.33), and level of outdoor contact (P = 0.25), in relation to wild boar observations, only geographical region showed a significant association. When the same parameters were investigated for association between geographical region (P = 0.65), main type of production (P = 0.58), farm size (P = 0.97) or level of outdoor contact (P = 0.88), to the closest distance where wild boar were observed, no significant associations were found.

The two outcome variables, wild boar observations and shortest distance to the observation of wild boars or their activities, showed a significant association (P < 0.01) when assessed.

The explanatory variable main type of production was significantly associated with the level of outdoor contact (P < 0.01), and farm size (P < 0.01). Likewise, in univariate analysis the explanatory variables farm size and level of outdoor contact was associated (P < 0.01), as was main type of production and geographical region (P < 0.1).

## Discussion

For the last decades, the wild boar population has been on the rise in Sweden and the rest of Europe [18]. Disease presence among wild boar populations represents a risk for disease introduction to domestic pigs. For ASF, the greatest risk for disease transmission from wild boars to domestic pigs is likely through indirect contact with the external environment [6, 7, 19] and the potential of indirect contact at Swedish pig farms is supported by this study. The fact that respondents to a large degree observed wild boar or their activities implies that, if these animals carry an infection, contamination of the immediate farm environment could occur with subsequent risk of disease transmission.

This study could not correlate the frequency of wild boar observations to any of the recorded farm characteristics, farm size, main type of production or level of outdoor access. However, the recorded presence of wild boar is associated with geographical region. Wild boars are, to a large extent, present close to Swedish commercial pig farms with 80% of the responding pig producers stating that they observed wild boar or wild boar activities in the vicinity of their farm at least once during the year. Although wild boars are shy and rarely observed directly, their presence is readily detected as rooting, sometimes with an addition of tracks or droppings. Farmers in the regions where wild boar are present are experienced in observing the signs of these animals and the risk of false positive responses to these questions may be regarded as low.

Overall, the distribution of production types and farm sizes represented in the responses reflect Swedish pig production. The recruitment for this survey, involving pig health advisory organisations, made it possible to reach the vast majority of Swedish pig producers and we believe that the results sufficiently reflect commercial pig farms in areas where wild boars are present.

Pigs raised commercially in Sweden are mostly kept indoors. Outdoor access is mainly seen on organic farms, which represented 2.6% of the slaughtered pigs in 2020 [20]. Some of the respondents with outdoor access for their pigs stated they had few pigs, indicating that they were not typical commercial holdings. While perimeter fencing around Swedish pig farms is rare, all pigs with outdoor access are required by law to be fenced in. Although fencing reduces the risk of direct contact with wild boar, this risk is not completely eliminated as wild boars may still break through or reach domestic pigs across fences. Double fences further reduce the risk of direct contact or fence breakthrough, but this study shows that double fencing is not used by all farmers. Four of the respondents stated use of naturally occurring open water sources for cleaning of pig houses, with only one also letting the pigs drink such water. Contamination of open water sources by infected wild boar might result in disease transmission if the concentration of the infectious agent is high enough in the water used in the pig house [21].

The majority of the questionnaire respondents who grew crops for pigs had observed wild boar damages in their fields. Hence, at least theoretically, indirect transmission of infectious agents from wild boars to domestic pigs via contaminated straw harvested from these fields is possible since straw is extensively used for bedding and enrichment in Swedish pig production.

Our results indicate that hunting and strategic baiting are the most prevalent mitigation strategies in use to avoid wild boar presence around pig farms, but responses stating doing nothing to control the wild boar population were also common. The response of not applying any strategies may reflect that not all producers are hunters or possess the hunting rights in the areas surrounding their farm, and also that some responding producers’ farms are located in areas where the wild boar is less common. Hunting abroad in areas where ASF is present in the wild boar population has been proposed as a risk of introducing the disease to Sweden. A few of the respondents indicated that they or their staff engaged in hunting abroad, which merits further investigation. Potential mitigation strategies included measures to draw wild boars away from the farm (strategic baiting), fencing to prevent them entering as well as reducing the population and hence the risk of unwanted visits. A combination of these strategies seems warranted but require collaboration between different actors (farmers, land owners, hunting rights owners, and hunters) in the same region.

Almost all farms with outdoor access responded that it would be possible to raise their pigs indoors in a disease outbreak situation where restrictions on outdoor access would be imposed. As the results of this study confirm the potential for indirect contact between wild boar and domestic pigs, this remains an important consideration for disease preparedness in Swedish pig production.

Not all participants answered all questions. The questions were grouped in sections on separate pages in the questionnaire, and missing answers were mainly seen in the end of sections, whereas the questions displayed at the top of each page were more often answered by all respondents. This can be partly explained by the layout, where questions located at the end of the section might not have been noticed before progressing. Still, most questions were answered by a majority of the respondents and the number of responses were sufficient for the analyses. The strong association between different farm characteristics is not surprising but prevented assessment of any single risk factor for wild boar presence in the farm vicinity. Nevertheless, it seems that most pig farms located in areas where wild boars are present will be at risk for indirect contact between wild boars and the domestic pigs. It is also important to keep in mind that the wild boar situation in Sweden is not static. Even though a farm currently may not experience wild boar contacts, the local wild boar abundance may rapidly change and require adaptation or deployment of mitigation strategies. Hunting activities in regions in which ASF is present among wild boars are also important for the risk of introduction of ASF to the wild boar population in Sweden. Other studies are currently investigating these aspects.

## Conclusions

The results of this study confirm that wild boars are present in close vicinity of commercial pig farms in Sweden, providing opportunities for contamination of the immediate farm environment should an infectious disease like ASF be present. Apart from geographical region, no other investigated potential risk factor was found to be associated with wild boar observations. Wild boar presence around pig farms calls for measures to mitigate direct and indirect contact between wild boar and domestic pigs and a need for deeper understanding of the wildlife/livestock interface to adjust measures accordingly.

### Supplementary Information

**Additional file 1: **Web questionnaire questions as PDF, translated to English from Swedish by authors.

## Data Availability

To ensure anonymity, the categorized version of the dataset regarding geographic location and farm size is available from the author on reasonable request.

## Declarations

### Prior publication

Data has not been published previously.
