# Supplementary material for: Understanding the interface between European wild boar (Sus scrofa) and domestic pigs (Sus scrofa domesticus) in Sweden through a questionnaire study
Source: Acta Vet Scand. 2023 Sep 22;65:40. doi: 10.1186/s13028-023-00705-x (PMC10515417; doi:10.1186/s13028-023-00705-x)
Supplement: Supplementary file 1 — Additional file 1: Web questionnaire questions as PDF, translated to English from Swedish by authors. [file 13028_2023_705_MOESM1_ESM.pdf]

Questions from an online questionnaire survey directed at commercial pig holders in Sweden regarding wild boar observations in the vicinity of their pig holding. The study was conducted in November 2019 to January 2020

First two digits in your farms postal code: ___
Main pig production of farm: □ Farrow-to-finish production; □ Piglet production; □ Finisher pig production; □ Live animals (Breeding/Gilts); □ Other, please specify: ___
Animal trade category: □ Conventional, indoor; □ Outdoor access; □ Specific Pathogen free (SPF); □ Other, please specify: __________
Approximate number of year-sows in production (SIP), per year: □ I do not keep sows; □ Number of sows (SIP): ___
Approximate number of finisher pigs produced yearly: □ I do not keep finisher pigs; □ Number of finisher pigs: _____
What pig housing alternatives are present at your farm: (Check all that apply) □ Conventional pen/group pen indoors; □ Pen/group pen in outdoor climate barn; □ Outdoor access with fence/electric fence; □ Other, please specify: ______________
Water source for pigs (drinking): □ Municipality water; □ Well; □ Open water source (lake/creek); □ Other, please specify: ________
Water source for cleaning of pig housing: □ Municipality water; □ Well; □ Open water source (lake/creek); □ Other, please specify: ________
In the last 12 months, have you had a suspected hybrid litter between wild boar and domestic pig: □ Yes; □ No
In the last 12 months, have you observed wild boar, or signs of wild boar activity (rooting/footprints/droppings) close to pigs with outdoor access? □ Not relevant, no pigs with outdoor access; □ No, no wild boar close to pigs with outdoor access; □ Yes. Please specify the distance (m): _____
In the last 12 months, have you observed wild boar, or signs of wild boar activity (rooting/footprints/droppings) close to pigs in outdoor climate barns? □ Not relevant, no pigs in outdoor climate barns; □ No, no wild boar close to pigs in outdoor climate barns; □ Yes. Please specify the distance (m): __
In the last 12 months, have you observed wild boar, or signs of wild boar activity (rooting/footprints/droppings) close to conventional indoor pig houses? □ Not relevant, no pigs in

conventional pig houses, indoor; □ No, no wild boar close to pigs in outdoor climate barns; □ Yes. Please specify the distance (m): __
Have you, this year (2019) had damage caused by wild boar in crops grown for use in pigs: (Multiple choices possible): □ I do not grow crops for use in pigs; □ No, no wild boar damage in my crops; □ Yes, in grain; □ Yes, in other types of crops. Please specify what crop: ___
In the last 12 months, have you observed wild boar, or signs of wild boar activity (rooting/foot prints/droppings) close to other buildings, not housing pigs, at your farm (Multiple choices possible) □ No, there is no wild boar in my area; □ No, wild boar is present in the area but they do not come close; □ Yes, by feed- or litter storage; □ Yes, by other buildings. Please specify: ___
Approximately how frequently do you observe wild boar or signs of wild boar activity (rooting, footprints, droppings) during each of the four seasons (spring, summer, autumn, winter). Question asked for each season, separately: □ Never; □ Very rarely, once during the season; □ Rarely, once monthly during the season; □ Often, every week during the season; □ Very often, daily, or close to daily, during the season.
What mitigation strategies do you use at your farm to avoid contacts between the wild boar and domestic pigs: (check all that apply): □ Perimeter fence surrounding the farm; □ Double fencing in pig pens; □ Hunting; □ Strategic use of feeding/baiting in other location; □ Nothing; □ Other, please specify; _____
In case of restrictions imposed by animal disease outbreak, could you for a period of a few months, keep all your pigs solely indoors? (Please disregard any certifications that require outdoor access): □ Not relevant, no pigs with outdoor access, my pigs are already kept indoors; □ Yes, pigs that are kept outdoors or have outdoor access can temporarily be housed solely indoors; □ No, please specify what prevents you from housing the pigs indoors (e.g. lack of building, lack of feeding/watering facilities): __
Do you hunt for wild boar: □ Yes, in Sweden only; □ Yes, in Sweden and abroad; □ Yes, only abroad; □ No
Does any of your staff in contact with the pigs hunt for wild boar: □ Yes, in Sweden only; □ Yes, in Sweden and abroad; □ Yes, only abroad; □ No; □ I do not know
